# Immunogenic Cell Death, DAMPs and Prothymosin α as a Putative Anticancer Immune Response Biomarker

**DOI:** 10.3390/cells11091415

**Published:** 2022-04-22

**Authors:** Anastasios I. Birmpilis, Antonios Paschalis, Apostolis Mourkakis, Panayiota Christodoulou, Ioannis V. Kostopoulos, Elina Antimissari, Georgia Terzoudi, Alexandros G. Georgakilas, Christina Armpilia, Panagiotis Papageorgis, Efstathios Kastritis, Evangelos Terpos, Meletios A. Dimopoulos, Hubert Kalbacher, Evangelia Livaniou, Maria-Ioanna Christodoulou, Ourania E. Tsitsilonis

**Affiliations:** 1 Section of Animal and Human Physiology, Department of Biology, School of Sciences, National and Kapodistrian University of Athens, Panepistimiopolis, Ilissia, 15784 Athens, Greece; abirmpilis@biol.uoa.gr (A.I.B.); apaschal@med.uoa.gr (A.P.); ivkostop@biol.uoa.gr (I.V.K.); 2 DNA Damage Laboratory, Physics Department, School of Mathematical and Physical Sciences, National Technical University of Athens, 15780 Zografou, Greece; alexg@mail.ntua.gr; 3 Medical Physics Unit, 1st Department of Radiology, School of Medicine, Aretaieion Hospital, National and Kapodistrian University of Athens, 11528 Athens, Greece; charbilia@med.uoa.gr; 4 Tumor Immunology and Biomarkers Laboratory, Basic and Translational Cancer Research Center, Department of Life Sciences, European University Cyprus, Nicosia 2404, Cyprus; am201795@students.euc.ac.cy (A.M.); pa.christodoulou@euc.ac.cy (P.C.); ea212000@students.euc.ac.cy (E.A.); 5 Laboratory of Health Physics, Radiobiology & Cytogenetics, Institute of Nuclear and Radiological Sciences and Technology, Energy and Safety, National Centre for Scientific Research “Demokritos”, Agia Paraskevi, 15310 Athens, Greece; gterzoudi@rrp.demokritos.gr; 6 Tumor Microenvironment, Metastasis and Experimental Therapeutics Laboratory, Basic and Translational Cancer Research Center, Department of Life Sciences, European University Cyprus, Nicosia 2404, Cyprus; p.papageorgis@euc.ac.cy; 7 Department of Clinical Therapeutics, School of Medicine, National and Kapodistrian University of Athens, 11528 Athens, Greece; ekastritis@med.uoa.gr (E.K.); eterpos@med.uoa.gr (E.T.); mdimop@med.uoa.gr (M.A.D.); 8 Interfaculty Institute of Biochemistry, Eberhard Karls Universität Tübingen, 72076 Tübingen, Germany; hubert.kalbacher@uni-tuebingen.de; 9 Institute of Clinical Anatomy and Cell Analysis, Eberhard Karls Universität Tübingen, 72074 Tübingen, Germany; 10 Immunopeptide Chemistry Laboratory, Institute of Nuclear and Radiological Sciences and Technology, Energy and Safety, National Centre for Scientific Research “Demokritos”, Agia Paraskevi, 15310 Athens, Greece; livanlts@rrp.demokritos.gr

**Keywords:** apoptosis, biomarker, bortezomib, γ-radiation, DAMP, doxorubicin, decapeptide proTα(100–109), immunogenic cell death, prothymosin alpha, regulated cell death

## Abstract

The new and increasingly studied concept of immunogenic cell death (ICD) revealed a previously unknown perspective of the various regulated cell death (RCD) modalities, elucidating their immunogenic properties and rendering obsolete the notion that immune stimulation is solely the outcome of necrosis. A distinct characteristic of ICD is the release of danger-associated molecular patterns (DAMPs) by dying and/or dead cells. Thus, several members of the DAMP family, such as the well-characterized heat shock proteins (HSPs) HSP70 and HSP90, the high-mobility group box 1 protein and calreticulin, and the thymic polypeptide prothymosin α (proTα) and its immunoreactive fragment proTα(100–109), are being studied as potential diagnostic tools and/or possible therapeutic agents. Here, we present the basic aspects and mechanisms of both ICD and other immunogenic RCD forms; denote the role of DAMPs in ICD; and further exploit the relevance of human proTα and proTα(100–109) in ICD, highlighting their possible clinical applications. Furthermore, we present the preliminary results of our in vitro studies, which show a direct correlation between the concentration of proTα/proTα(100–109) and the levels of cancer cell apoptosis, induced by anticancer agents and γ-radiation.

## 1. Introduction

Immunogenic cell death (ICD) is a type of regulated cell death (RCD), increasingly studied in recent years, due to its therapeutic implication in several diseases associated with immune system dysfunction. Key players in ICD are the danger-associated molecular patterns (DAMPs) [1,2,3], evolutionary conserved stress signals, recognized primarily by innate immune system receptors [4]. The immunogenicity of DAMPs characterizes ICD, rendering them potential prognostic, diagnostic clinical tools and/or possible therapeutic targets. Recent studies strongly suggest that the ubiquitous, multifaceted polypeptide, prothymosin alpha (proTα) and its immunoreactive fragment, the carboxy (C)-terminal decapeptide proTα(100–109), can act as DAMPs, highlighting new emerging roles for the two molecules [5]. Herein, we summarize the basic concepts of ICD, present the mechanisms underlying ICD induction, and describe the role of DAMPs in the most extensively studied ICD modalities. We also present the various functions of proTα and proTα(100–109), emphasizing on their role as immunostimulatory DAMPs, and provide evidence for their eventual use as biomarkers that could predict response to anticancer treatment.

## 2. Immunogenic Cell Death (ICD): Moving beyond the Classic Death Dipole Apoptosis/Necrosis

ICD defines various RCD processes which, upon stimulation by endogenous antigenic components from dying or dead cells, lead to an enhanced T cell-dependent immune response [1,2,6]. Immunogenicity derives from the synergy of these antigens with DAMPs (also termed alarmins), which act as adjuvants when secreted/excreted in the microenvironment of dying cells [1,2,3]. ICD is also characterized by the production of high levels of reactive oxygen species (ROS) and is highly related to endoplasmic reticulum (ER) stress, often resulting in an unfolded protein response (UPR) [7]. 

**Figure 1:**
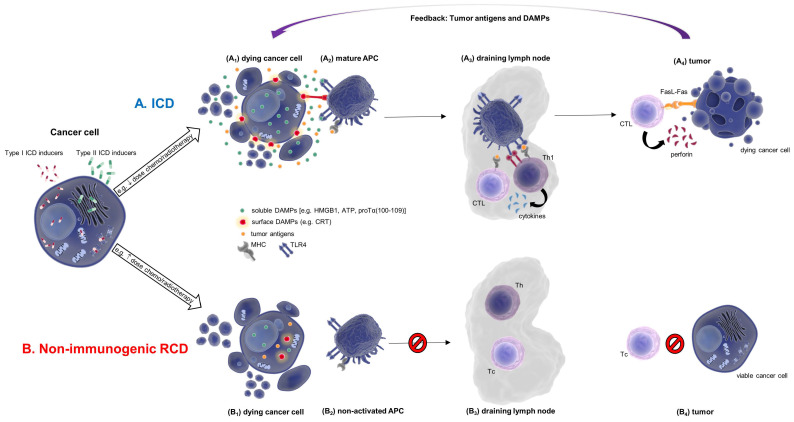
Differences in adaptive immune responses following induction of ICD and non-immunogenic RCD. (**A**) In ICD, type I ICD inducers trigger indirect ER stress; type II ICD inducers trigger direct ER stress. The dying cancer cell releases tumor antigens and DAMPs (e.g., HMGB1, ATP, proTα(100–109)), while “eat-me” signals (e.g., CRT) are exposed on the cell surface (A1). Maturation of APCs increases cancer antigen uptake and MHC class II and I are overexpressed (A2). Mature APCs traffic to the draining lymph node, produce Th1-polarizing cytokines and chemokines, and activate helper T (Th1) and cytotoxic T cells (CTLs) (A3). CTLs traffic to the tumor and, via the secretory (perforin) or the non-secretory (Fas-FaL) pathways, kill cancer cells (A4). The de novo released tumor antigens and DAMPs act as feedback signals and refuel the ICD cycle (arrow). (**B**) In non-immunogenic RCD, the dying cancer cell does not release tumor antigens and DAMPs (B1), APCs are not activated (B2), helper and cytotoxic T cell responses are not stimulated (B3), and cancer cells remain viable (B4).

For years, the common conception regarding the way by which cells die has been fallaciously limited to the dipole apoptosis/necrosis [3,8]. The apoptotic process, historically considered as solely representing RCD, is characterized by programmed morphological changes occurring in the apoptotic cell, including the initial DNA fragmentation and the subsequent formation of apoptotic bodies, i.e., membrane vesicles enwrapping intracellular material [9,10]. These changes often promote the recognition and engulfment of the shrunken apoptotic cell or smaller membranous portions thereof by phagocytes, in a non-immunologically-mediated manner [8,10,11]. Thus, apoptotic cell death has been considered as immunologically “silent” and, subsequently, immunologically tolerated [6,12,13,14]. On the other hand, necrosis and, later on, necroptosis, have been strongly associated with inflammation [6,8,10,11,15]. Nonetheless, scientific data clarified this long-held misconception and rendered some RCD types as potential inducers of adaptive immune responses [8,16]. Galluzzi et al. [17] and Kroemer et al. [18] elegantly described that the immunological outcome of RCD, i.e., whether immunogenic or tolerogenic, depends on the presence of antigens (antigenicity), on potent and immunostimulatory adjuvant-like signals (adjuvanticity) and on the “shaping” of an immune-permissive microenvironment. In the therapeutic management of tumors, treatment with low-dose chemotherapy (e.g., anthracyclins) or low-dose ionizing radiation (IR; e.g., γ-rays) induces ICD. The latter is associated with tumor-antigen shedding and translocation or release of DAMPs (e.g., calreticulin (CRT), high-mobility group box 1 protein (HMGB1), adenosine triphosphate (ATP)) by dying cells, which bind to innate immune receptors on antigen-presenting cells (APCs); the concomitantly released type I interferons (IFNs) and interleukin (IL)-1β modulate the microenvironment to support APC maturation and trafficking to the draining lymph nodes, where they dictate T cell activation and proliferation; tumor-reactive T cells further traffic to the tumor, rapidly eliminate cancer cells and ultimately, culminate in tumor antigen-specific immunological memory (Figure 1). On the contrary, apoptotic non-inflammatory RCD induced for example by high-dose chemotherapy or radiation, causes blebbing of the tumor-cell membrane, loss of tumor-antigen(s) and of DAMP secretion/excretion, impedes APC activation and secretion of pro-inflammatory cytokines, and consequently, inhibits the activities of effector T cells, finally leading to immunosuppression [19,20].

**Figure 2:**
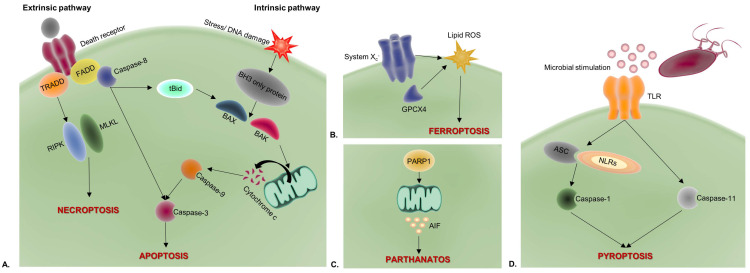
An overview of ICD mechanisms and key regulatory molecules. The main ICD modalities shown are apoptosis and necroptosis (**A**), ferroptosis (**B**), parthanatos (**C**), and pyroptosis (**D**). Arrows indicate the pathway flow and the relative regulatory molecules. AIF, apoptosis-inducing factor; ASC, apoptosis-associated speck-like protein containing a CARD; BAK, Bcl-2 homologous antagonist killer; BAX, Bcl-2-like protein 4; FADD, FAS-associated death domain protein; GPX4, glutathione peroxidase 4; MLKL, mixed lineage kinase domain-like pseudokinase; NLRs, NOD-like receptors; PARP1, poly(ADP-ribose) polymerase-1; RIPK, receptor-interacting protein kinase; ROS, reactive oxygen species; TLR, Toll-like receptor; and TRADD, tumor necrosis factor receptor type 1-associated death domain protein.

Unlike the accidental/necrotic cell death (ACD) caused by various physical, chemical, and mechanical cell injuries, RCD is elegantly controlled by a plethora of molecular signaling pathways. Well-characterized RCD modalities reported to stimulate immunogenic properties through the release of DAMPs are shown in Figure 2.

### 2.1. Apoptosis

Apoptosis is the most extensively studied type of RCD. An apoptotic cell undergoes a variety of rigorously programmed processes that affect its morphology, including the condensation and fragmentation of chromatin, the rupture of the nucleus, and a decrease of cellular volume and blebbing, which, finally, result in the formation of apoptotic bodies. Apoptotic bodies contain intracellular material and organelles and are eventually cleared by non-professional (e.g., macrophages) and professional (e.g., dendritic cells (DCs)) phagocytes, via a process known as efferocytosis [2,10]. Based on the stimulus that initiates the apoptotic cascade, apoptosis may be mediated by two distinct pathways, the extrinsic and the intrinsic [2,21,22]. 

The extrinsic or receptor-mediated pathway is initiated by the stimulation of death receptors, belonging to the tumor necrosis factor (TNF) family, such as CD95 (APO-1/Fas) or TNF-related apoptosis-inducing ligand (TRAIL), that activate caspase-8, the primary initiator of the caspase cascade. Caspase-8 is responsible for the direct cleavage of downstream effector caspases, such as caspase-3 [2,9,22,23]. 

The intrinsic or mitochondrial pathway is initiated by stress-induced signals, followed by the release and accumulation of apoptogenic, mitochondria-derived factors in the cytoplasm, such as cytochrome c, apoptosis-inducing factor (AIF), the second mitochondria-derived activator of caspase/direct inhibitor of apoptosis-binding protein with low pI (Smac/DIABLO), the serine protease high-temperature requirement A2 (HtrA2/Omi), and endonuclease G. Accumulation of cytochrome c in the cytoplasm triggers the formation of the cytochrome c/apoptotic protease-activating factor 1 (Apaf-1) apoptosome complex, which recruits and activates pro-caspase-9, subsequently resulting in the activation of caspase-3. Smac/DIABLO and HtrA2/Omi interact with and antagonize the inhibitor-of-apoptosis proteins (IAPs), whereas AIF and endonuclease G translocate to the nucleus, thus promoting DNA condensation [2,9,21,22,24].

Numerous studies have shown that apoptosis mediated by either the extrinsic or the intrinsic pathway can be immunogenic [25]. Albert et al. first observed that human DCs can efficiently present antigens derived from apoptotic monocytes previously infected with influenza A virus and stimulate major histocompatibility complex (MHC) class I-restricted CD8+ cytotoxic T lymphocytes (CTLs) [26]. It was further shown that protein cleavage, generated by activated caspases during apoptosis, facilitates antigen processing and cross-presentation by DCs [27,28]. At the same time, the release of DAMPs by apoptotic cells triggers immune responses. Specifically, tumor cells treated with doxorubicin (DX) and other anthracyclines, oxaliplatin (OXP) or IR, elicit anticancer immune responses in vivo and thus, grant protection to mice against tumor growth [16,28,29]. This procedure involves the release of various DAMPs by apoptotic cells, including CRT, members of the heat shock protein (HSP) family, such as HSP70 and HSP90, HMGB1 and ATP. These molecules are essential prerequisites for characterizing an RCD case as immunogenic [28]. The aforementioned data suggest that apoptotic cells may be immunogenic, whereas necrotic cells induce excessive inflammation, due to the massive release of DAMPs, but are incapable of eliciting potent CD8+ T cell responses [25,30].

### 2.2. Necroptosis

Necroptosis is a type of RCD, morphologically resembling necrosis. It is triggered by the stimulation of TNF receptors, such as TNF receptor 1 (TNFR1) and Fas [2,31], or pattern recognition receptors (PRRs), such as DNA-dependent activator of interferon-regulatory factors (DAI) and Toll-like receptors (TLRs) 3 and 4 [2,32]. Via signal transduction, the receptor-interacting protein kinase (RIPK) 1 is activated and RIPK3 is consequently recruited. RIPK3 further activates the mixed lineage kinase domain-like pseudokinase (MLKL), which promotes cell membrane breaching and cell death, with simultaneous spilling of intracellular content that contains pro-inflammatory cytokines and DAMPs [2]. The aforementioned DAMPs, together with the released cytokines and chemokines, render necroptotic cells immunogenic and thus, able to elicit CD8+ T cell-mediated responses, including potent anticancer responses [3,6], since necroptosis bypasses tumor cell resistance to apoptosis. 

### 2.3. Pyroptosis

Pyroptosis is an RCD modality initiated by intracellular and extracellular homeostatic perturbations, associated with the innate arm of immunity [9]. Specifically, it is triggered in response to pathogenic infections, e.g., with *Salmonella* spp. [33]. Similarly to necroptosis, pyroptotic cells present a necrotic morphology, characterized by plasma membrane rupture that results in the release of their cellular content [9,33]. In general, it occurs in phagocytes, such as macrophages, DCs and neutrophils, although it has been observed in other cell types as well. The mechanism of pyroptosis is strongly linked to the enzymatic activity of caspases, especially of caspase-1, and its activation associates with inflammasomes—cytosolic structures assembled by activated specific PRRs [34]. This activation results in the release of IL-1β, a pyrogenic cytokine that induces fever and recruits immune cells to the infected tissue, and IL-18, which conditionally promotes either T helper (Th) 1 or Th2 immune responses. Furthermore, as the pyroptotic process involves membrane breaching, it consequently leads to the release, among other intracellular components, of DAMPs, such as HMGB1, several S100 proteins, and IL-18α [33,34]. Strong evidence suggests that neutrophil pyroptosis may play a pivotal role in sepsis [35].

### 2.4. Ferroptosis

Ferroptosis also shares common morphological characteristics with necrosis and is triggered by cellular homeostatic disturbances, associated with impaired regulation of intracellular iron levels, leading to a lethal iron-dependent accumulation of lipid hydroperoxides [36,37]. This severe lipid peroxidation is associated with the release of immunostimulatory DAMPs, such as HMGB1, and cytokines, such as IL-1β and IL-18, by the ferroptotic cell, thus rendering the ferroptotic process immunogenic [38,39]. 

### 2.5. Parthanatos

Parthanatos is another form of RCD that features necrotic-like morphology. It is the result of severe/prolonged alkylating DNA damage and is driven by hyperactivation of a specific component of the DNA damage response. It is also involved in the pathogenesis of several conditions, such as ischemia-reperfusion injury, hypoxia, inflammation, myocardial infarction, glutamate excitotoxicity and Parkinson’s disease [9,40]. The key molecule implicated in the mechanism of parthanatos is poly(ADP-ribose) polymerase-1 (PARP-1), a nuclear protein that plays an important role in DNA repair, genomic stability, and transcription [40,41]. In cells facing excessive DNA damage, overactivation of PARP-1 eventually drives cells to RCD, as a result of the depletion of cellular energy, the mitochondrial release of AIF, and the production of excess poly(ADP-ribose) (PAR) polymers [40]. Activation of PARP-1 induces the release of immunogenic alarmins, primarily of HMGB1. 

Overall, based on this evidence, various RCD processes can likely be deemed as forms of ICD. Some additional death types include anoikis (an apoptotic RCD modality) [1,9,42,43], mitochondrial permeability transition (MPT)-driven necrosis [9], entotic cell death (entosis) [9,44], the neutrophil extracellular trap (NET) cell death or NETosis [9,44,45], lysosome-dependent cell death (LDCD) [9,44], autophagy-dependent cell death (ADCD) [9,44], autosis [9,44,46], alkaliptosis [44], and oxeiptosis [44], but their detailed analysis is beyond the scope of this review. Major types of ICD along with their morphological characteristics and immunologic profiles are briefly presented in Table 1, and regulatory molecules required for ICD induction and coordination of the process are comparatively shown in Figure 2.

## 3. Danger-Associated Molecular Patterns (DAMPs) Are Major Players in ICD

A common and crucial characteristic among the aforementioned ICD modalities is the release of DAMPs to the microenvironment of dead or dying cells. Their adjuvanticity is a prerequisite that accounts for triggering the stimulation of immune responses associated with RCD. Under normal conditions, DAMPs are located inside various cell compartments (e.g., nucleus, cytosol, ER, etc.) and are not implicated in immunological functions; nevertheless, upon elicitation of ICD mechanisms, they are often subjected to modifications, such as oxidation or proteolysis, and are eventually released or excreted extracellularly, adopting an immunogenic role [47]. 

Some known immunostimulatory DAMPs related to ICD are CRT [16], HMGB1, HSPs [28], ATP [47], and proTα/proTα(100–109) [5]. CRT is normally found in the ER, and its main role is the maintenance of calcium ion (Ca^2+^) homeostasis. Upon induction of ICD and prior to cell membrane breaching, CRT translocates to and is exposed on the cell membrane, where it acts as an “eat me” signal for phagocytes, such as DCs [28,48]. HSPs act in a similar, though less clarified way [48]. Contrarily, HMGB1, normally a nuclear protein responsible for DNA organization and transcription regulation, acts at later stages of the cell death process. Specifically, after leaking out of the ruptured cell membrane, HMGB1 has a dual function: on the one hand, it is captured by APCs and induces the production of proinflammatory mediators, such as TNF and IL-1β, while on the other, it signals via TLR2 and TLR4 and stimulates innate immune cells [49]. ATP has been reported to act through activation of the P2X purinoceptor 7 (P2RX7) on DCs, resulting in the formation of inflammasomes and, subsequently, the activation of caspase-1 [34]. The proposed mechanisms of action of proTα and its immunoreactive decapeptide proTα(100–109) are discussed in detail below. On the basis of the above, it is clear that DAMPs act diversely and at different stages of the ICD process. Of note, some DAMPs released during RCD are immunosuppressive, e.g., prostaglandin E2 (PGE2) and translationally-controlled 1 tumor protein, and in such cases, adaptive immunity is dampened [18]. 

## 4. Inducers of ICD and Their Types

In recent years, the development of novel therapeutic interventions to treat pathologies such as cancer, has shifted towards biological therapies, mostly of an immunological nature. The concept of driving altered cells to an elegantly regulated form of death, capable of eliciting in vivo anticancer immune cell-dependent responses, has shown promise. Specifically, it has been shown that increased HMGB1 translocation to the cytoplasm of tumor cells can serve as a predictive biomarker of better clinical outcomes in patients with locally advanced rectal cancer treated with neoadjuvant chemotherapy [50]; in patients with esophageal squamous cell carcinoma, high serum HMGB1 levels have been correlated with stronger tumor antigen-specific T cell responses and better clinical outcome after chemoradiation [51]. HSP70 has been correlated with prolonged progression-free survival in patients with glioblastoma [52], and increased CRT exposure on ovarian carcinoma cells was linked to Th1 effector polarization, cytotoxic cell activation, and superior disease outcome [53]. Thus, new therapeutic approaches have emerged or are currently under evaluation in clinical trials, aiming at exploiting the benefits of ICD induced by anticancer drugs or treatments (Table 2).

Depending on whether the stimulation of ICD is associated with an indirect or direct ER stress response, ICD inducers are classified into two distinct categories: type I and type II, respectively [48,54]. 

### 4.1. Type I ICD Inducers

Type I ICD inducers are molecules that indirectly induce ER stress responses, such as UPR, without targeting the ER itself; thus, they trigger ICD-associated danger signaling that is not linked with ROS production and ER stress [48,54] (Figure 1). Type I ICD inducers are chemical agents or radiation means that can kill tumor cells while concomitantly initiating an immunogenic signal transduction. Anthracyclines, like DX, mitoxantrone (MTX), and idarubicin (IDA) [15,48,54,55,56], various proteasome inhibitors, such as bortezomib (BTZ) and carfilzomib (CFZ) [8,15,55,56], OXP [15,48,54,56], shikonin [54] and IR, such as γ-radiation [56,57,58,59], are well-known type I ICD inducers. 

Anthracyclines are extensively used to treat various malignancies and are known to induce ICD in cancer cells in vitro. Specifically, human REH (lymphoblastic leukemia), OV90 (ovarian cancer), and DU145 (prostate cancer) cells, treated with DX or IDA for 12 and 24 h, showed increased expression of the DAMPs “eat me” signals HSP70, HSP90, and CRT, accompanied by their translocation to the cell surface. Furthermore, a significant increase of HMGB1 concentration was detected in cell supernatants of these cell cultures, 24 h post treatment [60]. In in vitro experiments on mouse CT-26 (colon cancer) cells, OXP stimulated the expression and translocation of CRT to the cell membrane, and the concomitant release of high levels of HMGB1, 4 h post treatment [61]. In vivo, vaccination of immunocompetent Balb/c mice with syngeneic OXP-treated CT-26 cells protected them against re-challenge with the same tumor cells [61]. The proteasome inhibitors BTZ and CFZ act through inhibition of the 26S proteasome, and the intracellular accumulation of misfolded and unfolded proteins, which further cause collateral ER stress, followed by UPR and subsequently ICD [8,62] and release of DAMPs. It has been shown that BTZ enhances the DC-mediated T cell response via promoting the exposure of HSP90 on the cell membrane of dying myeloma cells [63]. Shikonin is a phytochemical compound that can either trigger the extrinsic apoptotic pathway by stimulating death receptors followed by activation of caspase-8, or the intrinsic apoptotic pathway by activating the apoptosis regulator Bcl-2-like protein 4 (BAX), leading to cytochrome c release and activation of caspase-9 [64]. B16 mouse melanoma cells, cultured in the presence of shikonin for 24 h, exhibited elevated expression of HSP70, HSP90, CRT, and HMGB1 [55]. Apart from the aforementioned ICD-inducing drugs, Encouse et al. showed that mouse TSA (mammary carcinoma) cells, exposed to therapeutic IR doses in vitrο, were driven to ICD because they exhibited significant CRT translocation to the cell surface and elevated HMGB1 and ATP release [58]. IR can cause apoptosis via both the extrinsic and the intrinsic pathways, although it primarily acts through the latter. Specifically, it acts in a dose-dependent manner, causing double-strand DNA breaks (DSBs), single-strand breaks (SSBs) and/or DNA base damages. The inability of cells to repair the aforementioned types of DNA damage induces cellular stress which, in most cases, triggers the intrinsic apoptotic pathway that results in activation of caspase-9, and further, of the effector caspases-3 and -7, leading to ICD and DAMP release [59,65]. 

### 4.2. Type II ICD Inducers

Type II ICD inducers selectively target the ER, directly inducing ER stress responses, usually via the release of ROS [48,54] (Figure 1). Photodynamic therapy (PDT) has reportedly been associated with type II ICD induction, through the manipulation of photosensitizers, such as hypericin (Hyp) and oxygen to generate ROS; these trigger photo-oxidative (phox-ER) stress and subsequently URP-associated signaling pathways [66]. Hyp-PDT treatment induced ICD in human T24 (urinary bladder carcinoma) and mouse CT-26 cells. Furthermore, immature human DCs, when incubated in vitro with Hyp-PDT-treated T24 cells, matured and upregulated MHC class II expression. Release of DAMPs, such as CRT, by the dying Hyp-PDT-treated T24 cells was also observed [67]. Although type II ICD inducers act through a plethora of mechanisms, their common sole target is the ER. The ER stress response caused by various treatments (e.g., regimens that include BTZ, CFZ, sorafenib, etc.) leads to potent induction of ICD and elicits both innate and adaptive immune responses. 

### 4.3. Other ICD Inducers

Apart from the aforementioned binary classification of ICD inducers, a group thereof trigger a variety of concurrent effects and cannot be strictly classified in either of the two categories. An example is bleomycin (BLM), a complex of cytotoxic glycopeptide antibiotics that, similar to IR, causes DNA breaks by generating excess amounts of ROS and causing ER stress. BLM-induced ICD leads to the translocation of CRT to the plasma membrane of dying cells and the release of HMGB1 and ATP. In addition, it has been shown that BLM elicits CD8+ T cell-mediated responses, enhances the production of IFN-γ by T cells, and induces the expansion of forkhead box P3+ (Foxp3+) regulatory T cells via the secretion of transforming growth factor β by tumor cells [55,68].

## 5. The Novel DAMP proΤα: Characteristics and Role

ProΤα is a highly conserved acidic protein, ubiquitously expressed and detected in all mammalian cells and tissues. Initially isolated from rat thymus in 1984 by Nassos Haritos [69], proTα belongs to the family of immunoactive polypeptides known as thymosins. Human proTα is encoded by the *PTMA* gene (2q37.1) and comprises 109–110 amino acids [5]. Physiologically, it is a nuclear polypeptide [70], bears a nuclear localization signal (NLS) [71], and is not secreted, due to the absence of a relevant signal peptide sequence [5]. Almost 40 years of accumulated evidence support a dual role for proTα, both intracellularly and extracellularly; nevertheless, its mode of action remains controversial.

### 5.1. Intracellular Role of proTα

Intracellularly, proTα is involved in key cellular events, such as cell proliferation, survival, and apoptosis, as well as in the processes of DNA replication and transcription. It has been shown that cells with a high proliferative potential, such as cancer cells, overexpress the *PTMA* gene [72] and tumors are rich in proTα content [73]. Hence, proTα has been used as a marker of proliferation, correlated with tumor aggressiveness in various malignancies [72]. ProTα interacts with the linker histone H1, acting as a molecular chaperone. In the absence of proTα, H1 binds to nucleosomes and induces the condensation of euchromatin to heterochromatin [74]. ProTα regulates the transportation of H1 from and to chromatin [75] and thus leads, on the one hand, to the promotion of DNA replication, and on the other, to the formation of the cAMP-response element binding protein (CREB) – CREB-binding protein (CBP)–p300 complex, chromatin remodeling, and gene transcription [76]. ProTα has also been shown to protect cells from apoptosis via a mechanism that involves its interaction with Apaf-1, resulting in the inhibition of apoptosome formation and, by extension, of the apoptotic cascade [73,77]. Furthermore, proTα is also known to protect cells from oxidative stress caused by ROS, by inducing the nuclear translocation of NF-E2 related factor 2 (Nrf2) [78]. This complicated intracellular role of proTα bears a strong resemblance to the intracellular role of the prototype alarmin HMGB1. Similarly to proTα, HMGB1 is a ubiquitously expressed non-histone nuclear protein with chaperone activity mediated via its interaction with H1 [79]. This activity justifies the implication of HMGB1 in several cellular processes, such as the regulation of gene transcription [80], DNA replication [81], DNA repair [82], and apoptosis [79,83], clearly highlighting an almost identical intracellular profile to that of proTα.

### 5.2. Extracellular Role of proTα

Extracellularly, proΤα has been repeatedly shown to act as an immunomodulating agent, capable of eliciting potent immune responses, both in vitro and in vivo. The extracellular activity of proTα was first reported in 1986, when Pan et al. showed that the administration of proTα to immunosuppressed mice protected them from opportunistic infections [73,84]. This observation was further confirmed by a plethora of in vitro studies, that focused in principle on the tumor-related properties of proTα. Human T cells treated with proTα produced high levels of IL-2, as well as increased expression of the IL-2 receptor on their surface [73,85,86]. In cancer patient-derived peripheral blood lymphocytes, proTα augmented the cytotoxicity of natural killer (NK) and lymphokine-activated killer (LAK) cells [72,73], and induced the production of cytokines, by concomitantly reducing the levels of PGE2 and increasing the levels of IL-2 [73]. In addition, proTα upregulated the expression of MHC class II on human APCs, DCs and cancer cells, that otherwise expressed low or no MHC class II molecules [87]. We were the first to reveal that the core of the immunomodulatory properties of proTα is located at the C-terminal fragment 100–109 of the molecule, namely, proTα(100–109) [88]. Notably, the decapeptide proTα(100–109) also contains the NLS, i.e., the sequence TKKQKT (proTα(100-105)) [89]. We also showed that in vitro, proTα or its immunoreactive decapeptide induce the maturation of monocyte-derived immature DCs and, in the presence of tumor antigens and autologous immune cells, enhance the ability of DCs to stimulate Th1-type responses and activate tumor antigen-reactive CTLs [90]. Moreover, both proTα and proTα(100–109) can stimulate neutrophils from patients with cancer, increasing their phagocytic capacity and ROS production, and thus, enhancing their cytotoxicity versus cancer cells [91]. 

In our recent publication, we confirmed the anticancer activity of the two peptides in vivo. Specifically, the combined therapeutic administration of melanoma-derived peptides with proTα or proTα(100–109) delayed tumor growth in mice previously inoculated with syngeneic melanoma cells and increased their survival through the enhancement of antitumor-reactive T cell responses [92]. By immunohistochemistry, we detected higher numbers of T cells infiltrating the tumor bed in mice treated with the peptides compared to controls and by cytokine profiling, we showed a Th1 polarization of the immune response. 

These observations, along with the now undisputed fact that both proTα and proTα(100–109) signal via ligating TLR4 [90,93,94], allowed us to comprehend the immunomodulatory effects of the two peptides. The scenario we propose on the mechanism of action of proTα and proTα(100–109) is that initially the peptides ligate TLR4 on the surface of TLR4-expressing APCs, e.g., DCs, inducing their maturation; activated APCs efficiently uptake antigens (e.g., tumor-derived peptides), overexpress MHC class I and II, secrete proinflammatory cytokines and chemokines, and elicit a potent Th1-polarized response. Within this cytokine/chemokine-mediated permissive microenvironment, anticancer cytotoxic effectors are efficiently primed [92]. In other words, both peptides exhibit the adjuvanticity trait of DAMPs, providing robust danger/activation signals to the innate arm of immunity, and form a tumor microenvironment favoring effector cell activation. Further to their comparable intracellular role, the extracellular activity of proTα and proTα(100–109) which involves signaling via TLRs, reveals additional significant similarities to the extracellular functions of HMGB1: HMGB1 signals via TLRs 2 and 4 [49] to elicit both innate or adaptive immune responses [49,95]; induces the production of proinflammatory cytokines, such as TNF, IL-1β and IL-8 [82]; triggers oxidase activation in neutrophils [96]; whereas in several cases, HMGB1 has been shown to orchestrate a specific anticancer response, through the activation of APCs via TLR2/TLR4 signaling and tumor cell elimination by tumor-specific T cells [95,97]. Another remarkable similarity between the aforementioned molecules is that the source of their immunoreactivity relies on certain peptide fragments, namely proTα(100–109) for proTα, and the small peptides Hp-16, Hp-31 (A box) and Hp-91 (B Box) for HMGB1 [98,99,100]. Overall, despite their structural differences, proTα and HMGB1 exhibit extraordinary resemblances to both their intra- and extracellular activity.

### 5.3. Evidence Supporting That proTα Acts as Alarmin—Proposed Role of proTα(100–109)

The key mechanism for bridging the intracellular and extracellular role of proTα is the fragmentation of the protein that occurs during apoptosis [101]. As proposed, during RCD and mainly during apoptosis, proTα translocates from the nucleus to the cytoplasm, where it is primarily cleaved at D^99^ and at a number of secondary sites. The principal enzyme, responsible for the apoptosis-mediated truncation of proTα, is effector caspase-3, although caspase-7 is also likely implicated, as previously shown in vitro [101]. Cleavage at D^99^ leads to the generation of the immunoreactive decapeptide proTα(100–109). Subsequently, the main peptidic “body” proTα(1-99), presumably unable to traverse the cell membrane due to its charge, either remains in the cytoplasm and is degraded, or is eventually exposed at the cell membrane where it acts as an “eat-me” signal. Concomitantly, proTα(100–109), either in its monomeric or in a polymeric form, as it can acquire a β-sheet conformation [89], is released extracellularly via an unknown mechanism, where it exerts its immunomodulatory effects [5]. ProTα(100–109), supplementary to intact proTα which is likely excreted during cell necrosis, can act as a DAMP. Given this series of events and taking into account the aforementioned similarities between both the intracellular and extracellular roles of proTα and HMGB1, we assume that proTα and its immunoreactive peptide can be classified as DAMPs. Moreover, depending on the mode of cell death induced, we reason that during apoptosis or other RCD modalities, proTα is truncated and proTα(100–109) is released in the microenvironment of the dying cells, whereas, during necrosis, intact proTα, and possibly proTα(100–109), are uncontrollably spilled from the ruptured cell membrane. In both cases, immune responses are triggered [5]. This has been experimentally shown in an in vivo murine model of sepsis. Mouse innate immune cells infected with *Klebsiella pneumoniae* strains were driven to either massive apoptotic or necrotic death, followed by a relevant release of solely proTα(100–109) or both intact proTα and proTα(100–109), respectively. In the first case, RCD causes the gradual generation and release of proTα(100–109), and thus a controlled immune response is initiated; on the contrary, necrotic death results in a burst of intact proTα, and maybe of proTα(100–109), extracellularly, resulting in extensive and excessive inflammation, immune system overactivation, and, finally, septic shock [102]. Overall, in cases of massive cell death, whether regulated or accidental, and depending on the type of cell death, the DAMPs proTα(100–109) and/or proTα is/are released, act as adjuvants and elicit immune responses via TLR4-expressing phagocytes, such as macrophages, monocytes, mature DCs, and neutrophils. Particularly in cancer, both molecules trigger adaptive immunity by recruiting immune cells, such as DCs, CD8+ T, and NK cells, into the tumor microenvironment, orchestrating Th1-type responses, complemented by the activation of highly effective cytotoxic effectors. While the use of proTα and its immunoreactive peptide as immunotherapeutic agents has been conceived and is currently being studied, based on these recent observations, the potential of using proTα and proTα(100–109) as diagnostic tools or predictive biomarkers of the type of cell death induced after anticancer treatment, has become prominent. 

### 5.4. Detecting and Quantifying proTα and proTα(100–109)

An efficacious biomarker must fulfill two general prerequisites: highly sensitive detectability and accurate quantification. We have developed an in-house, competitive ELISA for proTα(100–109), using high affinity-purified polyclonal antibodies (Abs), produced in immunized rabbits. The method features high specificity versus various biomolecules, including α-thymosins, such as thymosin α1 (Τα1), and high sensitivity along with a wide-range calibration curve (0.1 ng/mL to 10 μg/mL) [103]. An ELISA with a narrower working range (1 ng/mL to 1 μg/mL) for proTα has also been developed recently, using chicken polyclonal Abs [104]. Since the chicken Abs might also recognize the N-terminal regions of the polypeptide [104] while the rabbit Abs raised against the C-terminal fragment proTα(100–109) may also recognize intact proTα [103], discrimination between proTα and proTα(100–109) is not quite feasible. Nonetheless, the two methods of detection, ideally in an elegant combination, would be a valuable basic tool to further comprehending the role of both peptides, proTα and proTα(100–109), as alarmins in various pathological conditions such as cancer, infections, sepsis, and autoimmune diseases. For example, considering that proTα(100–109) is the immunoreactive site of the molecule, N-terminal peptides and intact proTα could initially be removed from assessed samples using the polyclonal Abs from the chicken egg yolk, and the depleted samples containing only the decapeptide could subsequently be screened with the rabbit anti-proTα(100–109) ELISA; another suggestion would be to remove the N-terminal reactive Abs from the polyclonal chicken egg yolk preparation, and form a sandwich ELISA, using as capture Abs the rabbit (anti-proTα(100–109)) Abs and the purified chicken (anti-proTα) Abs as detection Abs. Such a format could enable us to distinguish the extracellular release of proTα and/or proTα(100–109) during ICD. 

To further assess the diagnostic potential of proTα(100–109) [103], we ran a series of preliminary in vitro experiments to reveal whether the concentration of proTα(100–109) in cancer cell culture supernatants correlates with the percentages of early apoptotic cells. Human cancer cell lines were driven to apoptosis after exposure to various ICD inducers (i.e., DX, BTZ, or γ-radiation), commonly used in anticancer regimens. We selected representative ICD inducers from different classes, DX as a model chemotherapeutic, BTZ as a targeted therapy inducer, and γ-irradiation representative of radiotherapy [18]. 

**Figure 3:**
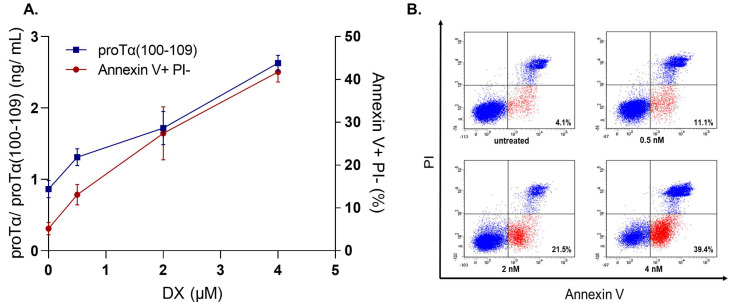
The levels of proTα(100–109) correlate with the percentages of early apoptotic MCF-7 human breast cancer cells treated with doxorubicin (DX). (**A**) An overlay chart, showing the concentration of the peptide as determined in MCF-7 culture supernatants (left axis) versus the percentages of early apoptotic (Annexin V+ PI−) MCF-7 cells (right axis). MCF-7 cells were treated with DX (0.5, 2 and 4 μM) for 48 h at a cell density of 1 × 10^6^ cells/mL. Values are means ± SD from three individual experiments performed. (**B**) Representative dot plots from one experiment with MCF-7 cells treated with DX. Cells were stained with Annexin V/PI and analyzed using BD FACSDiva software. Numbers in the low right quadrats show the percentages of early apoptotic cells.

**Figure 4:**
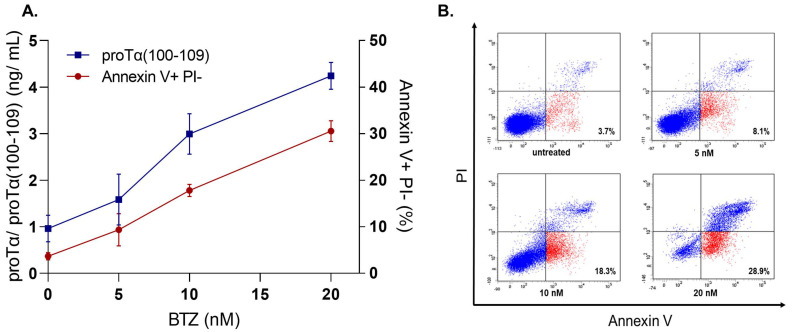
The levels of proTα(100–109) correlate with the percentages of early apoptotic H929 human myeloma cells treated with bortezomib (BTZ). (**A**) An overlay chart, showing the concentration of the peptide as determined in H929 culture supernatants (left axis) versus the percentages of early apoptotic (Annexin V+ PI−) H929 cells (right axis). H929 cells were treated with BTZ (5, 10, and 20 nM) for 72 h at a cell density of 1 × 10^6^ cells/mL. Values are means ± SD from three individual experiments performed. (**B**) Representative dot plots from one experiment with H929 cells treated with BTZ. Other details are as in the legend of Figure 3.

**Figure 5:**
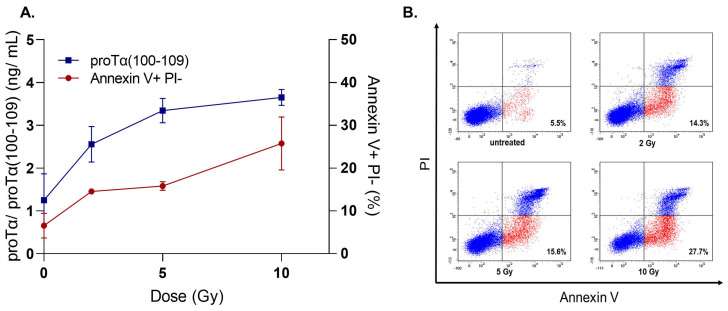
The levels of proTα(100–109) correlate with the percentages of early apoptotic H929 cells exposed to γ-radiation. (**A**) An overlay chart, showing the peptide’s concentration as determined in H929 culture supernatants (left axis) versus the percentages of early apoptotic (Annexin V+ PI−) H929 cells (right axis). H929 cells were exposed to γ-radiation of 2 Gy, 5 Gy, and 10 Gy and analyzed 72 h post irradiation. Values are means ± SD from three individual experiments performed. (**B**) Representative dot plots from one experiment with H929 cells exposed to γ-radiation. Other details are as in the legend of Figure 3.

We determined both the percentages of apoptotic cells (by flow cytometry following staining with FITC-Annexin V Apoptosis Detection Kit with Propidium Iodide (PI); BioLegend) and the concentration of proTα(100–109) in cell culture supernatants. The experiments were conducted οn adherent cells (MCF-7, breast cancer) and cells growing in suspension (H929, multiple myeloma), to simulate the use of the respective ICD inducers in patients with cancer. Specifically, MCF-7 cells (1 × 10^6^ cells/mL) were cultured with various concentrations of DX (0.5–4 μM) for 48 h. As shown in Figure 3, in untreated MCF-7 cells the percentage of early apoptotic cells was 5.16%, whereas a dose-dependent gradual increase was observed in DX-treated cells (13.08, 27.38, and 41.71% for 0.5 μM, 2 μM, and 4 μM DX, respectively). A similar gradual increase was also observed in proTα(100–109) concentration (0.862 ng/mL for untreated and 1.31–2.63 ng/mL for DX-treated cells). H929 showed a similar pattern when treated with BTZ (5–20 nM) for 72 h: a concomitant increase in both early apoptotic cell percentages (3.67% in untreated versus 30.53% in 20 nM BTZ-exposed) and proTα(100–109) levels (0.96 ng/mL in untreated versus 4.24 ng/mL in 20 nM BTZ-exposed) was observed (Figure 4). H929 cells were also driven to apoptosis after γ-irradiation (2−10 Gy) [56,57,58,59]. Specifically, H929 cells were vertically irradiated via a ^60^Co GammaCell 220 Irradiator (Atomic Energy of Canada Ltd., Ottawa, Canada) at room temperature, with a dose rate of 1 Gy/min, determined with a portable dosimeter placed in proximity with the cells. Next, cells were placed on ice for 30 min to prevent DNA repair, incubated at 37 °C for 72 h, harvested, and analyzed by flow cytometry. The results (Figure 5) confirmed our previous findings with DX and BTZ, showing a gradual increase in both apoptotic cell levels (6.53 to 25.73%) and proTα(100–109) concentration (1.25 to 3.65 ng/mL) as the radiation dose increased.

Based on these preliminary results, it would be interesting to determine proTα(100–109) in the peripheral blood of cancer patients treated with ICD inducers. As the release of proTα(100–109) from dying cells is an early apoptotic event [93], high levels of the decapeptide in blood within the first few days after therapy initiation could be a useful predictive biomarker for the early selection of responders to a given treatment. 

## 6. Conclusions

The various forms of RCD and their mechanistic insights, although not fully understood, are increasingly studied for their potential involvement in the treatment of several diseases. Among them, it is prominent that ICD bears a promising potential. In pathologies such as cancer, malignant cells develop various mechanisms to evade immune surveillance, often leading to the rapid exacerbation of the disease. In such cases, the induction of ICD could be deemed as an efficient countermeasure to the escape strategies of cancer cells. As previously highlighted, a common characteristic among all ICD modalities is the release of DAMPs. As DAMPs are immunomodulating agents capable of stimulating immune responses, their clinical significance is dual; they can either be used, due to their adjuvanticity, as therapeutic means to improve patient outcome, or as diagnostic tools and biomarkers to evaluate treatment efficacy. Accumulating evidence shows that proTα and its C-terminal peptide proTα(100–109), reportedly acknowledged for their immunoreactive properties, are members of the DAMP family, along with other known molecules such as HMGB1, CRT, and HSPs. 

The use of DAMPs as therapeutic agents is currently being evaluated, showing promising results. In cancer, which is an extensively studied field for the potential clinical applications of DAMPs, proTα and proTα(100–109) have been shown to elicit potent anticancer immune responses in vitro and in vivo, either as a standalone therapy or in combination with other treatments, e.g., peptide vaccines [85,90,91,92,105]. CRT has also shown anticancer properties in numerous in vitro and in vivo studies; however, its use as a therapeutic agent bears complications, albeit its efficacy may be improved once its mechanisms of action are elucidated [106]. The non-protein DAMP ATP was shown to delay the growth of various cancer types [107], while its intravenous administration increased the survival rate of patients with pre-terminal cancer [108]; however, like CRT, it has been associated with a series of side effects [109]. Vice versa, targeting DAMPs, such as HMGB1 and HPSs, with specific blockers can play a vital role in treating inflammatory diseases, such as sepsis, ischemia, myocardial infarction, etc. [110,111]. Provided that many ICD inducers, e.g., BTZ and CFZ, with established clinical efficacy and safety, have been authorized and are administered as anticancer drugs, and taking into consideration that DAMPs are potent agents with high adjuvanticity, the therapeutic potential of proTα and proTα(100–109) could be a new topic of study. 

On the other hand, the diagnostic and prognostic capabilities of DAMPs are gaining attention. The emerging correlation between the concentration of extracellular proTα/proTα(100–109) (i.e., in the serum of certain patient (sub)groups), and the levels of massive, immunogenic apoptotic death renders the peptides potential biomarkers with a plethora of potential clinical applications. The detection and quantification of the free peptide levels in the serum of cancer patients undergoing an ICD-inducing treatment could potentially correlate with patients’ responses to therapy, and by extension, predict the therapeutic efficacy of a specific regimen. Similarly, proTα(100–109) or intact proTα, as well as other DAMPs like HMGB1 and HSPs, can play a pivotal role in the differential diagnosis and prognosis of inflammatory and autoimmune diseases, such as sepsis and arthritis [102,110,111,112]. Furthermore, several clinical studies, completed or on-going, have been/are being conducted on the detection of DAMPs as ICD markers via various methods, e.g., flow cytometry, ELISA, etc. The use of HMGB1, CRT, and HSPs as biomarkers of ICD detection is clinically relevant in several pathological conditions, including cancer, sepsis, autoimmune diseases and acute or chronic inflammation (Table 2). The ICD-ICD inducer-DAMP axis is the cutting edge of immunotherapy, since the manipulation of its mechanisms has promising and unlimited capabilities. As major components of this axis, the alarmins proTα and proTα(100–109) have shown potential. Although additional research is required to fully comprehend the mechanistic pathways of ICD and further link the extracellular and intracellular role of α-thymosins in the context of ICD and its various forms, it would be of interest to assess the functions of proTα and proTα(100–109) as DAMPs, as well as their use as therapeutic agents, diagnostic tools, and/or predictive biomarkers, in a variety of immune-associated diseases and conditions, such as inflammation, autoimmunity, and cancer. 

## Figures and Tables

**Table 1:** The classification and characteristics of various cell death modalities. Important regulators mediating each death type are listed in the last column.

Cell Death Modality	Classification	Morphological Characteristics	Immunologic Profile	Regulators
Necrosis	ACD	cell swelling; DNA fragmentation; membrane rupture; loss of cell organelles	Tolerogenic/immunogenic	None
Apoptosis	RCD	cell shrinkage/rounding; nuclear condensation/fragmentation; nuclear membrane rupture; membrane blebbing; apoptotic body formation	Tolerogenic/immunogenic	Death receptors, BAX, BAK, AIF, caspases 2, 3, 6, 7, 8, and 9
Necroptosis	RCD	cell/mitochondrial swelling; membrane rupture; chromatin condensation; loss of cell organelles	Immunogenic	TLRs, TCR, RIPK1, RIPK3, MLKL
Pyroptosis	RCD	cell swelling; membrane permeabilization/rupture; DNA condensation/ fragmentation	Immunogenic	CASP1, CASP11, GSDMD, NLRs, ALRs
Ferroptosis	RCD	mitochondrial shrinkage; reduced mitochondrial cristae; mitochondrial membrane rupture	Immunogenic	System XC−, GPX4, TFRC, ACSL4, LPCAT3, ALOX15, GLS2, DPP4, NCOA4, BAP1, BECN1, PEBP1, CARS, VDAC2/3, RAB7A, HSP90, ALK4/5
Parthanatos	RCD	chromatin condensation; DNA fragmentation; membrane rupture; inconsistent mitochondrial membrane; no apoptotic body formation	Immunogenic	PARP-1, AIFM1, MIF, OGG1
Anoikis	RCD	cell shrinkage/rounding; nuclear condensation/fragmentation; nuclear membrane rupture; membrane blebbing; apoptotic body formation; detachment from substrate/other cells	Tolerogenic/immunogenic	Death receptors, BAX, BAK, AIF, caspases 2, 3, 6, 7, 8, and 9
MPT-driven necrosis	RCD	similar to necrosis; loss of mitochondrial inner membrane impermeability; mitochondrial membrane dissipation/breakdown	Immunogenic	CYPD (PPIF)
Entotic cell death (Entosis)	RCD	cell-in-cell formation	Tolerogenic/immunogenic	RhoA, ROCKI/II, E-cadherin, α-catenin, actomyosin, LC3, ATGs
Neutrophil extracellular trap cell death (NETosis)	RCD	membrane rupture; nuclear membrane dissolvement; chromatin decondensation/release	Tolerogenic/immunogenic	NOX4, PAD4, ELANE, MMP, MPO, ELANE, MMP, MPO
Lysosome-dependent cell death (LDCD)	RCD	lysosome/plasma membrane rupture	Immunogenic	BECN1, Na^+^/K^+^-ATPase, AMPK, Ras-like protein A
Autophagy-dependent cell death (ADCD)	RCD	vacuolization (large intracellular vesicles); enlargement of cell organelles; depletion of cell organelles	Immunogenic	UKL1, PI3KIII, ATGs, LC3
Autosis	RCD	enhanced cell-substrate adherence; ER fragmentation/breakdown; cell swelling; chromatin condensation	Immunogenic	Na+/K+-ATPase
Alkaliptosis	RCD	similar to necrosis	Immunogenic	IKBKB, NF-κB
Oxeiptosis	RCD	similar to apoptosis	Tolerogenic	KEAP1, PGAM5, AIFM1

ACD, accidental cell death; ER, endoplasmic reticulum; MPT, mitochondrial permeability transition; RCD, regulated cell death.

**Table 2:** Clinical trials evaluating ICD markers in various pathologies.

Identifier	Pathological Condition	DAMP(s)	Aim of Investigation	Status
NCT02921854	Cancer/non-small cell lung cancer	HMGB1, HSP70, CRT, HSP90	Detectability of ICD markers in the serum of patients post high-dose radiotherapy alone or concurrent cisplatin-doublet therapy and radiotherapy to access induction of anticancer immune responses.	Completed
NCT03581695	Pediatric pulmonary hypertension	HMGB1	HMGB1 levels in pediatric patients with pulmonary hypertension	Recruiting
NCT04837391	Postoperative cognitive dysfunction	HMGB1	Relationship between postoperative cognitive dysfunction and brain injury biomarkers in geriatric urologic oncology patients via measuring HMGB1 levels	Recruiting
NCT03986736	Tissue injury and rhabdomyolysis after major trauma	HMGB1	Correlation between the levels of HMGB1 and the degree of injury	Recruiting
NCT03741738	Autoimmuno diseases/Vitiligo	HMGB1	HMGB1 as a biomarker for predicting the severity of Vitiligo, by measuring apoptosis levels of melanocytes	Completed
NCT04080453	Sepsis/septic shock	HMGB1	Correlation of HMGB1 levels with platelet activation	Recruiting
NCT02914756	Sepsis/ Severe sepsis or septic shock at the ICU	HMGB1	HMGB1 levels in sepsis patients for weeks after recovery from severe sepsis/septic shock; association of prolonged HMGB1 levels in plasma with cognitive impairment in patients recovering from severe sepsis/septic shock	Completed
NCT03535441	Hemorrhagic shock (HS)	HMGB1	Determination of the levels of HMGB1-mediated inflammation in the serum of patients with HS	Completed
NCT03346018	Tuberculosis/ Sarcoidosis	HSP70	Establishment as a biomarker for the differential diagnosis of tuberculosis and sarcoidosis	Recruiting
NCT04614441	Certain types of lung disease	HMGB1, HSP27	Assessment of levels in patients with lung disease	Recruiting
NCT04787770	Diabetic atherosclerosis	HSP90	Assessment of the correlation between HSP90 levels and diabetic atherosclerosis	Completed
NCT05007444	Cancer/ Breast cancer	HMGB1, CRT, ATP	Assessment of the efficacy of the P2Et extract in ICD induction	Not yet recruiting
NCT01637532	Cancer/ Recurrent ovarian cancer	HMGB1, CRT	Assessment of the efficacy of carbo/doxorubicin/tocilizumab/ Peg-Intron combination in ICD induction	Completed

## Data Availability

Not applicable.

## Abbreviations

ACD: accidental cell death; ADCD, autophagy-dependent cell death; AIF, apoptosis-inducing factor; Apaf-1, apoptotic protease-activating factor 1; APC, antigen-presenting cell; ATP, adenosine triphosphate; BAX, Bcl-2-like protein 4; BLM, bleomycin; BTZ, bortezomib; CBP, CREB-binding protein; CFZ, carfilzomib; CREB, cAMP-response element binding protein; CRT, calreticulin; CTL, cytotoxic T lymphocyte; DAI, DNA-dependent activator of interferon-regulatory factors; DAMP, danger-associated molecular pattern; DC, dendritic cell; DSB, double-strand break; DX, doxorubicin; ER, endoplasmic reticulum; HMGB1, high-mobility group box 1; HSP, heat shock protein; HtrA2/Omi, high-temperature requirement protein A2; Hyp, hypericin; IAP, inhibitor-of-apoptosis protein; IDA, idarubicin; ICD, immunogenic cell death; IL, interleukin; IR, ionizing radiation; LAK, lymphokine-activated killer; LDCD, lysosome-dependent cell death; MHC, major histocompatibility complex; MLKL, mixed lineage kinase domain-like pseudokinase; MPT, mitochondrial permeability transition; MTX, mitoxantrone; NET, neutrophil extracellular trap; Nrf2, NF-E2 related factor 2; NK, natural killer; NLS, nuclear localization signal; OXP, oxaliplatin; P2RX7, P2X purinoreceptor 7; PAR, poly(ADP-ribose); PARP-1, poly(ADP-ribose) polymerase-1; PDT, photodynamic therapy; PGE2, prostaglandin E2; Phox, photo-oxidative; proTα, prothymosin α; PRR, pattern recognition receptor; RCD, regulated cell death; RIPK, receptor-interacting protein kinase; ROS, reactive oxygen species; Smac/DIABLO, second mitochondria-derived activator of caspase/direct inhibitor of apoptosis-binding protein with low pI; SSB, single-strand break; TLR, Toll-like receptor; TNF, tumor necrosis factor; TNFR1, TNF receptor 1; TRAIL, TNF-related apoptosis-inducing ligand; Tα1, thymosin α1; UPR, unfolded protein response.
